# Efficacy and safety of oxaliplatin-based chemotherapy as first-line treatment in elderly patients with metastatic colorectal cancer: a meta-analysis

**DOI:** 10.3389/fonc.2025.1567732

**Published:** 2025-04-07

**Authors:** Shaoqing Fan, Zeming Zhao, Haiqian Wang, Handong Wang, Wenbo Niu

**Affiliations:** ^1^ Department of General Surgery, The Fourth Hospital of Hebei Medical University, Shijiazhuang, Hebei, China; ^2^ Department of General Surgery, The Second Hospital of Hebei Medical University, Shijiazhuang, Hebei, China; ^3^ Department of Nursing, The Fourth Hospital of Hebei Medical University, Shijiazhuang, Hebei, China

**Keywords:** elderly, first-line treatment, metastatic colorectal cancer, meta-analysis, oxaliplatin

## Abstract

**Purpose:**

The global burden of colorectal cancer (CRC) continues to rise, with elderly populations disproportionately affected. Despite oxaliplatin’s established role in first-line metastatic CRC (mCRC) therapy, its clinical utility in older adults remains debated due to concerns over efficacy, toxicity, and survival outcomes. This meta-analysis evaluates the therapeutic benefits and risks of oxaliplatin-based regimens in elderly patients with mCRC, with emphasis on tumor response, survival endpoints, and treatment-related toxicities.

**Methods:**

We systematically reviewed PubMed, Web of Science, Cochrane Library, and Chinese databases (CNKI, Wan Fang) through November 2024 for randomized controlled trials (RCTs) comparing oxaliplatin-based chemotherapy to non-oxaliplatin regimens in patients aged ≥65 with mCRC. Outcomes included overall survival (OS), progression-free survival (PFS), objective response rate (ORR), complete response (CR), partial response (PR), disease control rate (DCR), and grade 3–4 adverse events. Data were pooled using random- or fixed-effects models in STATA 14.0 based on heterogeneity (I² statistic). Subgroup analyses explored heterogeneity sources, including chemotherapy combinations (e.g., bevacizumab, panitumumab).

**Results:**

Seven RCTs (1,839 patients) met inclusion criteria. Oxaliplatin significantly improved tumor response rates versus control regimens: ORR (OR 2.18, 95% CI 1.75–2.72; *P*<0.001), CR (OR 2.57, 1.11–5.97; *P*=0.028), and PR (OR 1.69, 1.28–2.22; *P*<0.001). No significant survival benefit was observed for OS (HR 0.97, 0.86–1.08; *P*=0.58) or PFS (HR 0.90, 0.79–1.01; *P*=0.07), though trends favored oxaliplatin. Grade 3–4 neutropenia (RR 1.84, 1.32–2.57), diarrhea (RR 2.01, 1.45–2.78), and sensory neuropathy (RR 3.12, 1.98–4.91) were more frequent with oxaliplatin. Subgroup analysis attributed DCR heterogeneity (I²=66%) to regimen differences, with reduced variability in bevacizumab/pantiumumab-combined subgroups.

**Discussion:**

This analysis demonstrates oxaliplatin’s capacity to enhance tumor response in elderly mCRC patients, potentially alleviating symptoms and improving quality of life. However, the absence of significant survival gains underscores the complex interplay between tumor biology and therapeutic resistance. Mechanistically, chemotherapy-driven clonal selection may favor residual resistant subpopulations, as evidenced by liquid biopsy studies linking tumor evolution to disease progression. While toxicity profiles were manageable, the elevated risk of neurotoxicity and myelosuppression necessitates vigilant monitoring in this vulnerable cohort.

**Conclusion:**

Oxaliplatin-based first-line therapy provides clinically meaningful tumor response improvements in elderly mCRC patients, though survival advantages remain elusive. Treatment decisions should balance response benefits against toxicity risks, prioritizing individualized strategies informed by geriatric assessments and molecular profiling. Future trials must integrate biomarker-driven approaches (e.g., ctDNA monitoring, RAS/RAF stratification) to optimize therapeutic precision in aging populations.

## Introduction

Globally, the incidence of colorectal cancer (CRC) has been on a steady rise, and similar trends are observed in China (1). According to the 2022 report on the burden of malignant tumors in China, colorectal cancer ranks among the top in both incidence and mortality (2). Liver metastasis is the primary factor that significantly reduces patient survival (3), and patient age also has a considerable impact on the prognosis of this disease (4, 5). For patients with metastatic colorectal cancer (mCRC), surgical resection is the only curative option; however, only approximately 25% of patients are eligible for radical surgery (6). Factors such as the number, size, and distribution of metastases, along with advanced patient age, are the main barriers to performing radical surgery (7, 8).

For patients who are ineligible for radical surgery, the first-line treatment options commonly include systemic chemotherapy regimens, such as FOLFOX, FOLFIRI, CapeOX, or FOLFOXIRI (9, 10), as well as novel immune checkpoint inhibitors like pembrolizumab, nivolumab, and envafolimab (11). However, these approaches still do not provide patients with an optimal survival time or quality of life. Notably, oxaliplatin use may result in liver damage, potentially leading to vascular injury, hepatic sinusoidal obstruction syndrome, and nodular regenerative hyperplasia (12, 13).

Due to eligibility criteria for chemotherapy trials, many studies exclude older patients, resulting in a lack of age-specific research data. The management of metastatic colorectal cancer (mCRC) in elderly patients presents distinct challenges, largely due to age-related physiological changes and comorbidities that impact treatment tolerance and efficacy. Despite the growing prevalence of mCRC in this population, elderly patients are often underrepresented in clinical trials, limiting the generalizability of standard treatment recommendations (14). This exclusion is particularly concerning given that older adults frequently present with complex clinical profiles, including a higher burden of comorbidities (e.g., cardiovascular disease, diabetes, and renal impairment), polypharmacy, and diminished physiological reserve, all of which can influence treatment outcomes (15).Age-related declines in renal and hepatic function, for instance, can alter the pharmacokinetics and toxicity profiles of chemotherapeutic agents such as oxaliplatin, necessitating dose modifications or alternative regimens (16). Additionally, the substantial heterogeneity within the elderly population complicates treatment decision-making. Chronological age alone is an inadequate predictor of treatment tolerance and outcomes, as functional status, frailty, and cognitive function vary widely among individuals (17). To address this variability, comprehensive geriatric assessment (CGA) tools, including the Cancer and Aging Research Group (CARG) toxicity score and the Chemotherapy Risk Assessment Scale for High-Age Patients (CRASH) model, have been developed to predict chemotherapy-related toxicities and guide treatment selection. However, their prospective validation in elderly mCRC patients receiving oxaliplatin-based therapy remains limited (18). Furthermore, striking an optimal balance between efficacy and toxicity is particularly challenging in this population. While aggressive treatment approaches may enhance tumor response, they also increase the risk of severe adverse events, potentially compromising quality of life and overall survival. Given these complexities, individualized treatment strategies incorporating CGA and biomarker-driven approaches are crucial to optimizing outcomes in elderly mCRC patients.

Existing studies have shown that the use of oxaliplatin in adjuvant chemotherapy for elderly patients with stage II/III colorectal cancer does not improve prognosis (18). In the context of first-line treatment for metastatic colorectal cancer (mCRC), the efficacy and safety of oxaliplatin-based chemotherapy in elderly patients remain inconclusive. Although oxaliplatin-based regimens have been associated with improved tumor response rates, including objective response rate (ORR), complete response (CR), partial response (PR), and disease control rate (DCR), their impact on overall survival (OS) and progression-free survival (PFS) in elderly patients remains uncertain (19). This discrepancy may be largely driven by the higher incidence of treatment-related toxicities, such as neurotoxicity, hematologic toxicity, and hepatotoxicity, which can necessitate dose reductions, lead to early treatment discontinuation, and ultimately compromise quality of life (20). Notably, oxaliplatin-induced neurotoxicity, a well-recognized dose-limiting adverse effect, affects approximately 15–20% of elderly patients and can severely impair daily functioning and treatment adherence, further complicating long-term clinical outcomes (21). Early studies failed to demonstrate significant benefits of adding oxaliplatin to chemotherapy regimens in this population (22). However, these studies were often limited by small sample sizes, which hindered the ability to draw definitive conclusions. To date, no systematic review or meta-analysis has been conducted to address this issue.

With the global population aging and the incidence of colorectal cancer (CRC) on the rise, optimizing treatment strategies for elderly patients with metastatic CRC (mCRC) has become increasingly urgent. This patient population presents unique clinical challenges, including a higher burden of comorbidities, polypharmacy, and diminished physiological reserves, all of which can impact treatment tolerance and therapeutic outcomes (16). A comprehensive evaluation of the efficacy and safety of oxaliplatin-based chemotherapy in elderly mCRC patients is therefore critical to informing evidence-based clinical decision-making and enhancing patient care. To address this need, the present study conducts a systematic review and meta-analysis of existing literature, with a particular focus on survival outcomes, tumor response rates, and treatment-related toxicities in this vulnerable population.

## Materials and methods

### Search Strategy

A systematic search of published literature was conducted using PubMed, Web of Science, Cochrane Library, China National Knowledge Infrastructure, and Wan Fang databases, covering publications from database inception through November 1, 2024. No language restrictions were applied in this meta-analysis.

The keywords searched were ((Oxaliplatin)or (1,2-Diamminocyclohexane(trans-1)oxolatoplatinum(II)) or (L-OHP Cpd) or (Platinum(2+) ethanedioate (1R,2R)-1,2-cyclohexanediamine (1:1:1)) or (Oxalato-(1,2-cyclohexanediamine)platinum II) or (Oxaliplatin, (SP-4-2-(1R-trans))-isomer) or (Oxaliplatine) or (1,2-Diaminocyclohexane Platinum Oxalate) or (1,2 Diaminocyclohexane Platinum Oxalate) or (Platinum(II)-1,2-cyclohexanediamine Oxalate) or (Cis-oxalato-(trans-l)-1,2-diaminocyclohexane-platinum(II)) or (ACT 078) or (ACT-078) or (ACT078) or (Oxaliplatin, (SP-4-3-(cis))-isomer) or (Eloxatine) or (Eloxatin) or (Oxaliplatin, (SP-4-2-(1S-trans))-isomer)) and ((Colorectal Neoplasms) or (Colorectal Neoplasm) or (Neoplasm, Colorectal) or (Neoplasms, Colorectal) or (Colorectal Tumors) or (Colorectal Tumor) or (Tumor, Colorectal) or (Tumors, Colorectal) or (Colorectal Cancer) or (Cancer, Colorectal) or (Cancers, Colorectal) or (Colorectal Cancers) or (Colorectal Carcinoma) or (Carcinoma, Colorectal) or (Carcinomas, Colorectal) or (Colorectal Carcinomas)) and ((elderly) or (older)) and ((Metastase) or (Metastases) or (Metastasis) or (Metastases, Neoplasm) or (Metastasis, Neoplasm) or (Neoplasm Metastases) or (Unresectable)).

### Selection of studies

The inclusion criteria were as follows: (1) Age ≥65 years; (2) Eastern Cooperative Oncology Group (ECOG) performance status of 0–2; (3) histopathologically confirmed colorectal cancer; and (4) imaging-confirmed metastatic lesions.

The exclusion criteria were as follows: (1) non-randomized controlled trials; (2) control group consisting of a placebo or no treatment; and (3) duplicate studies or studies with data that could not be independently extracted.

Two reviewers independently screened the titles and abstracts of all identified studies according to the search strategy, excluding those that did not meet the inclusion criteria. Discrepancies during screening were resolved by discussion, with a third reviewer consulted in cases of disagreement. Two independent reviewers evaluated the characteristics of the final included studies, summarizing details such as the first author’s name, year of publication, treatment protocol, number and gender of cases, median age of patients, number of rectal/colon cases, and research quality.

### Quality assessment

The quality of the included studies was assessed and graded using the modified Jadad scale (23), with scores of 1–3 indicating poor quality and scores of 4–7 indicating high quality.

### Data Extraction and Analysis

Clinical outcomes were extracted, and a joint analysis was performed using STATA 14.0 software (StataCorp LP). Heterogeneity was quantified with the I² statistic, where I² ≥ 50% indicated significant heterogeneity. For studies with I² ≥ 50%, outcome proportions and their 95% confidence intervals (CIs) were calculated using a random-effects model, whereas a fixed-effects model was used for I² < 50%. Sensitivity analysis, using a one-by-one elimination method, and subgroup analyses were conducted to investigate sources of heterogeneity. A P-value of < 0.05 was considered statistically significant.

## Results

### Search results

**Figure 1:**
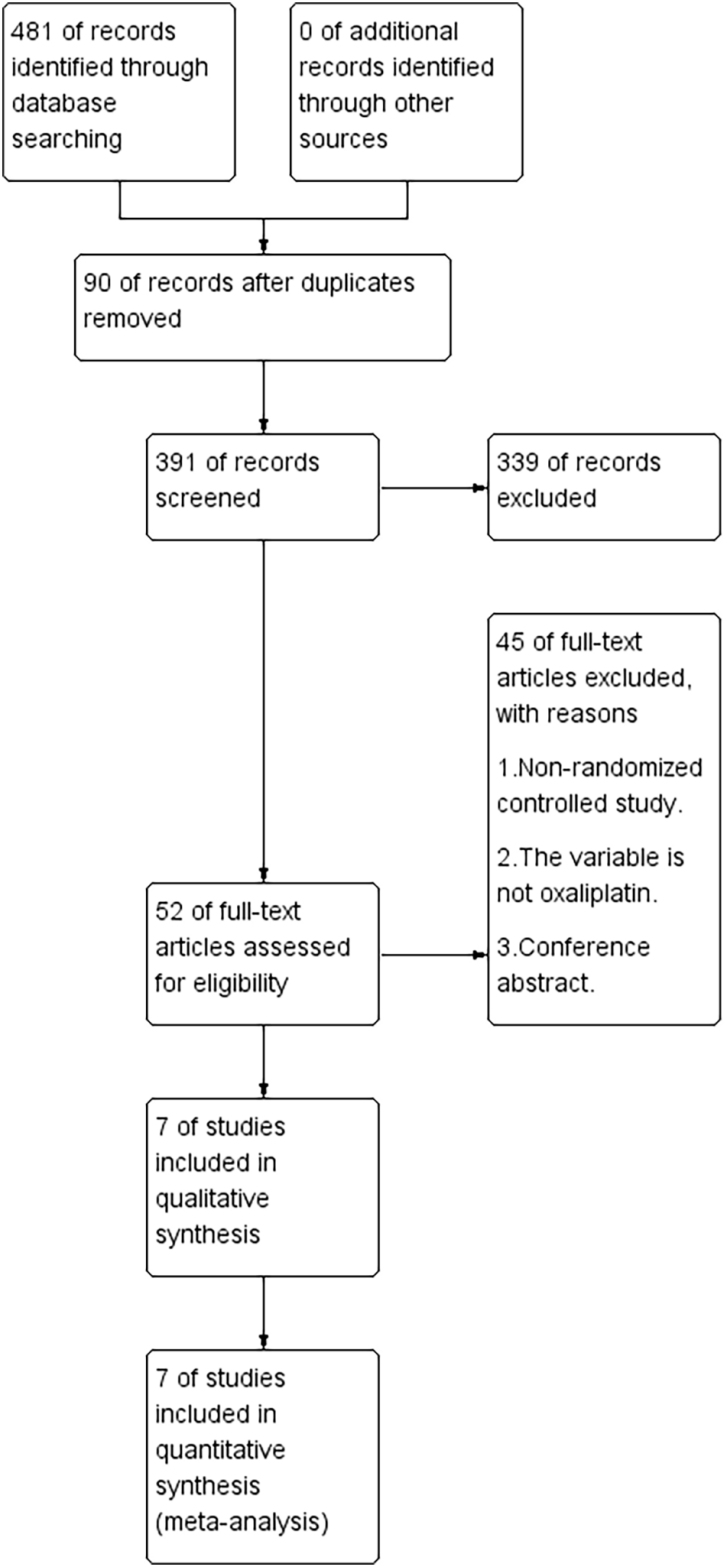
Flow diagram of the study selection process.

**Figure 2:**
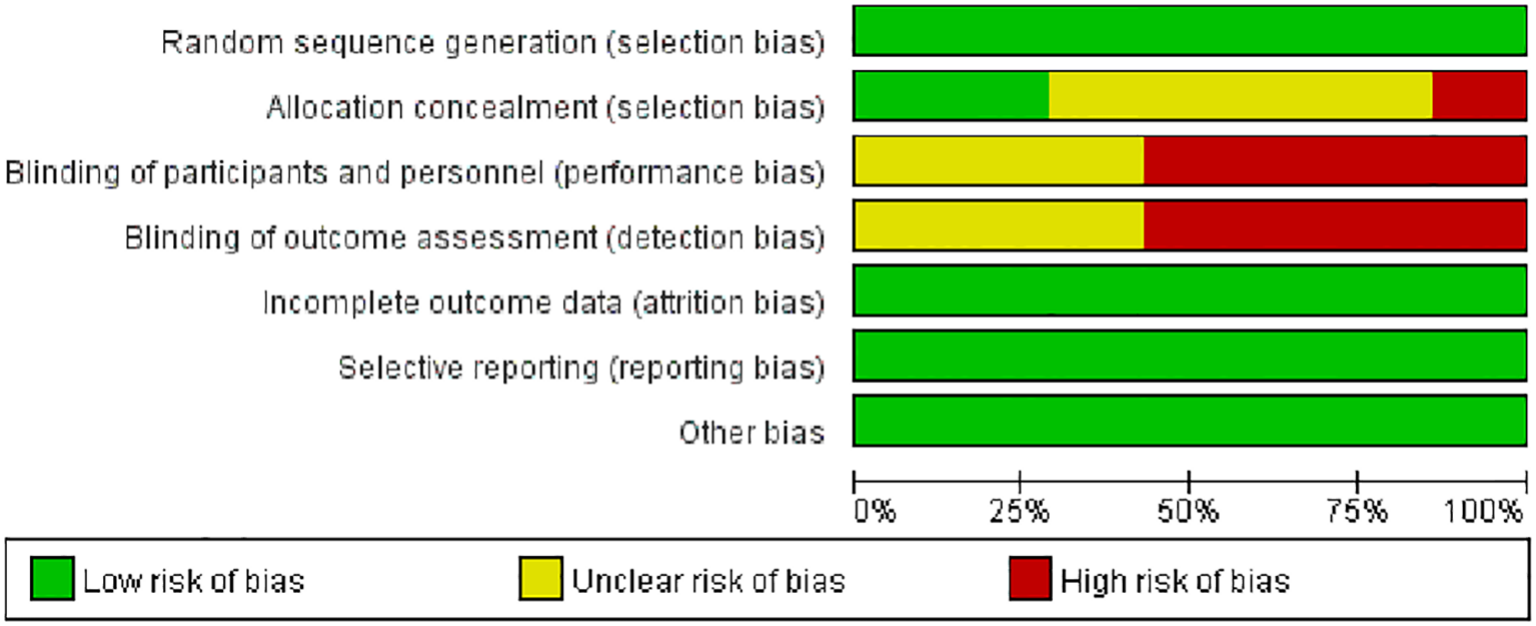
Jadad quality evaluation form.

A total of 481 articles were retrieved, with 90 identified as duplicates. After screening titles and abstracts, 391 articles were excluded based on the inclusion and exclusion criteria. After reviewing the full texts of the remaining studies, seven studies (22, 24–29) met the criteria and were included (**Figure 1**). Based on the Jadad scale, all seven studies were of high quality (**Figure 2**).

In total, 1,839 patients with metastatic colorectal cancer from the seven studies were included in this meta-analysis, with 910 patients in the treatment group and 929 in the control group. The characteristics of all patients are summarized in **Table 1**.

**Table 1:** Study information and patient characteristics.

First author	Year	Treatment protocol	Number of patients(Male/Female)	Median age (years)	Rectum/Colon	Research Quality	(Refs.)
Souglakos	2006	FOLFIRI	146(82/61)	66	36/110	4	(22)
		FOLFOXIRI	137(76/61)	66	37/100		
Vamvakas	2010	FOLFIRI	146(82/64)	72	36/110	4	(24)
		FOLFOXIRI	137(76/61)	72	37/100		
Seymour	2011	5-FU/Capecitabine	230(141/89)	74	56/174	4	(25)
		5-FU/Capecitabine + Oxaliplatin	229(137/92)	75	64/165		
Hong	2013	Capecitabine	40(23/17)	71	24/16	4	(26)
		Capecitabine + Oxaliplatin	40(22/18)	72	30/10		
Inada	2022	5-FU/Capecitabine + Bevacizumab	151(83/68)	68	54/97	5	(27)
		5-FU/Capecitabine + Bevacizumab + Oxaliplatin	149(91/58)	70	41/108		
Lonardi	2023	5-FU + LV + Panitumumab	91(60/31)	77	28/63	4	(28)
		mFOLFOX + Panitumumab	92(56/36)	77	32/60		
Takashima	2024	5-Fu + Bevacizumab	125(70/56)	80	47/78	5	(29)
		5-Fu + Bevacizumab + Oxaliplatin	126(65/60)	79	42/84		

### Efficacy Assessment

Primary outcomes

**Figure 3:**
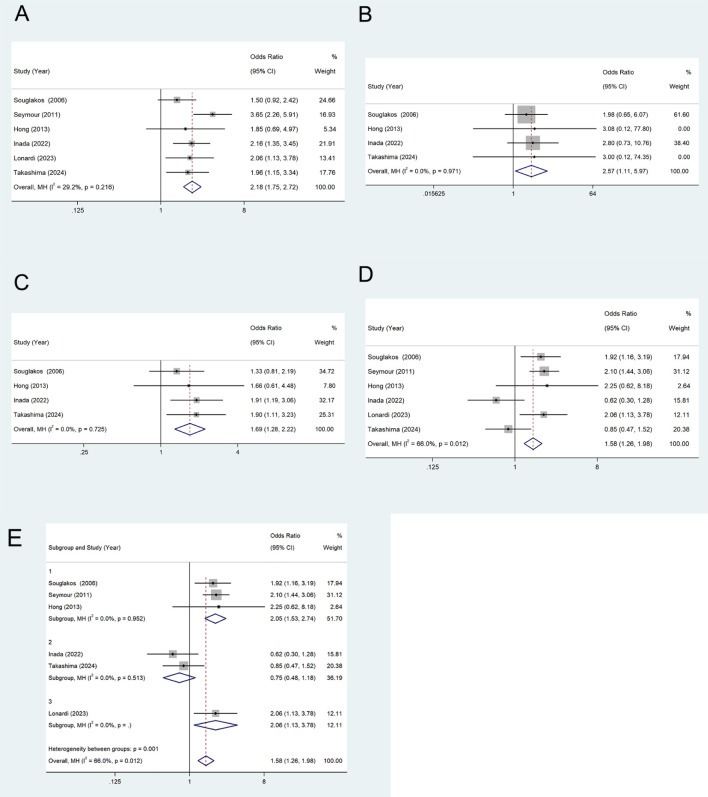
ORR **(A)**, CR **(B)**, PR **(C)**, DCR **(D)** and subgroup analysis **(E)** of Oxaliplatin treatment in elderly patients with metastatic colorectal cancers.

ORR, CR, PR and DCR. Six studies (22, 25–29) reported data on Objective Response Rate(ORR). The pooled odds ratio (OR) for all included patients was 2.18 (95% CI, 1.75–2.72, P=0.00, I²=29.2%), indicating no significant heterogeneity among studies (**Figure 3A**), thus a fixed-effects model was applied. Four studies (15, 19, 20, 22) reported data on Complete Response(CR) (**Figure 3B**) and Partial Response(PR) (**Figure 3C**), with ORs of 2.57 (95% CI, 1.11–5.97, P=0.028, I²=0.0%) and 1.69 (95% CI, 1.28–2.22, P=0.00, I²=0.0%), respectively, showing no significant inter-study heterogeneity. All results were robust and reliable. Six studies provided data on Disease Control Rate (DCR), with a pooled OR of 1.58 (95% CI, 1.26–1.98, P=0.078, I²=66%), indicating high heterogeneity. The sensitivity analysis did not show an obvious decrease in heterogeneity. A random-effects model was applied, and the result was not statistically significant (P>0.05) (**Figure 3D**). A subgroup analysis based on chemotherapy regimens was conducted, categorizing the studies into three groups: chemotherapy alone, chemotherapy combined with Bevacizumab, and chemotherapy combined with Panitumumab. The results indicated a significant reduction in heterogeneity (**Figure 3E**), suggesting that differences in chemotherapy regimens may contribute to the observed heterogeneity.

**Figure 4:**
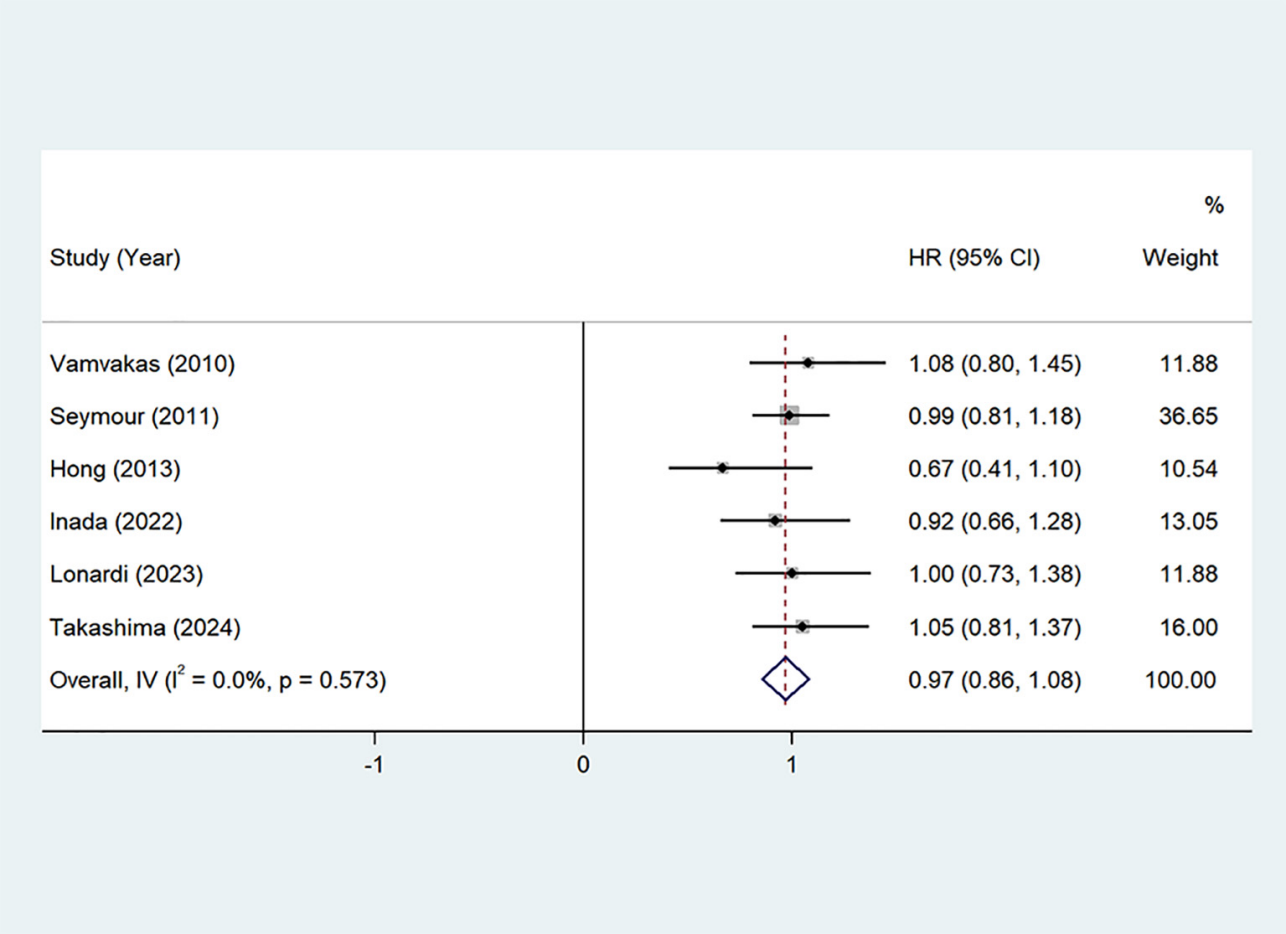
OS of Oxaliplatin treatment in elderly patients with metastatic colorectal cancers.

OS: Seven studies (22, 24–29) provided Overall Survival (OS) data. The pooled hazard ratio (HR) from these studies was 0.97 (95% CI: 0.86–1.08, P=0.00, I²=0.0%), indicating no significant heterogeneity. A fixed-effects model was applied, as shown in **Figure 4**. These findings suggest that the addition of oxaliplatin may contribute to an extension in OS, although the impact was not statistically significant.

**Figure 5:**
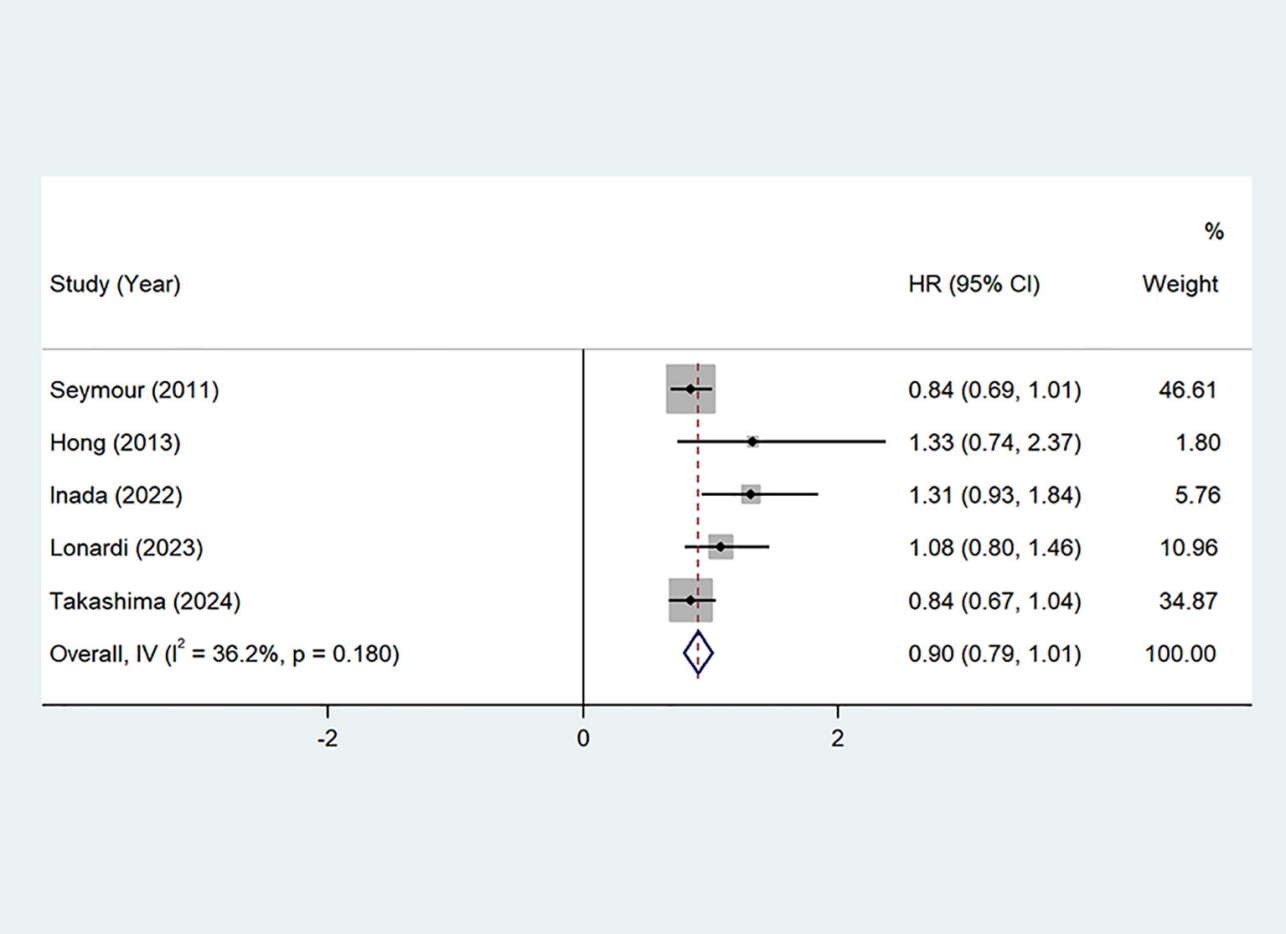
PFS of Oxaliplatin treatment in elderly patients with metastatic colorectal cancers.

PFS: Seven studies (22, 24–29) provided Progression-Free Survival (PFS) data. The pooled hazard ratio (HR) was 0.90 (95% CI: 0.79–1.01, P=0.00, I²=36.2%), with no significant heterogeneity. A fixed-effects model was applied, as shown in **Figure 5**. These results suggest that the addition of oxaliplatin may potentially prolong PFS, although the effect was not statistically significant.

### Secondary outcomes

**Figure 6:**
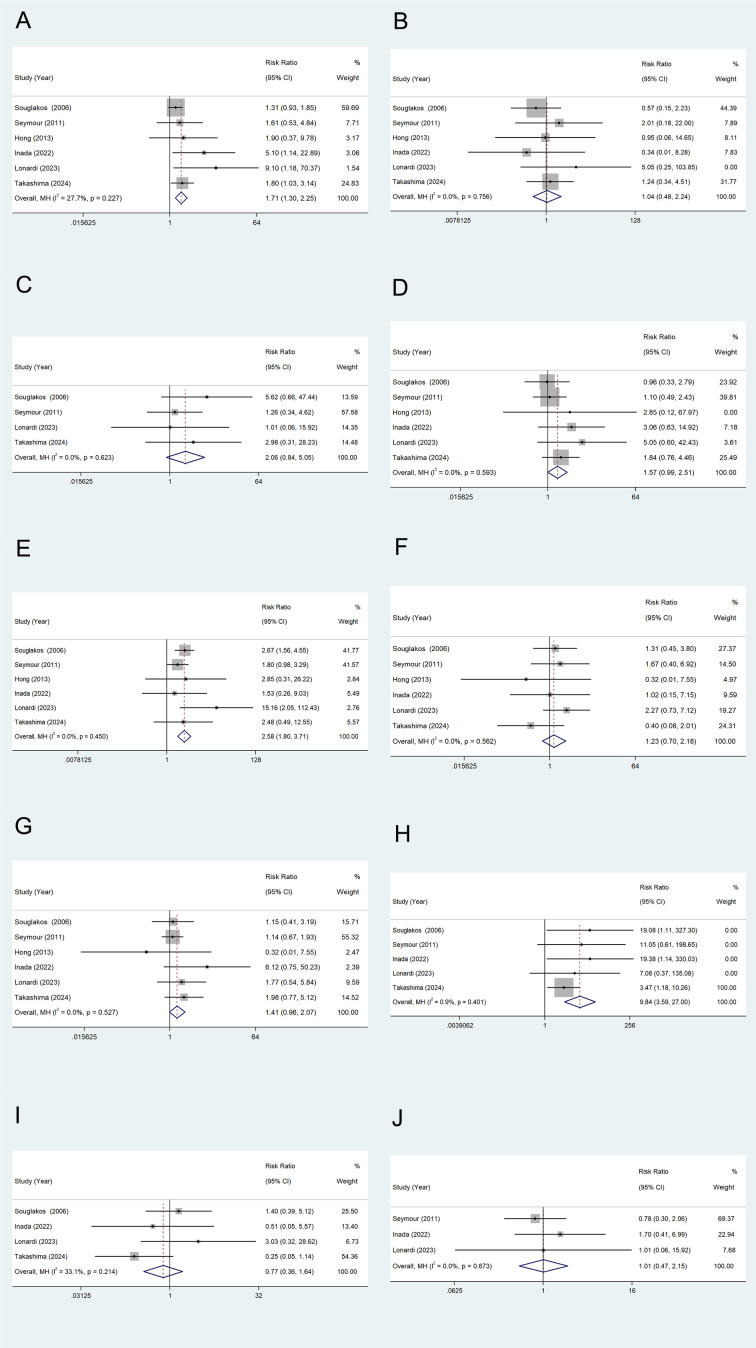
TEAEs of Oxaliplatin treatment in elderly patients with metastatic colorectal cancers. Neutropenia **(A)**, Thrombocytopenia **(B)**, Anemia **(C)**, Nausea/Vomiting **(D)**, Diarrhea **(E)**, Stomatitis **(F)**, Fatigue **(G)**, Sensory neuropathy **(H)**, Hand-foot syndrome **(I)**, and Anorexia **(J)**.

TEAEs: Six studies reported adverse events (TEAEs) in metastatic colorectal cancer patients treated with combined oxaliplatin. Most adverse events were grade 1 and 2, with only a few cases classified as grade 3 or 4. Common adverse reactions included neutropenia (**Figure 6A**), thrombocytopenia (**Figure 6B**), anemia (**Figure 6C**), nausea/vomiting (**Figure 6D**), diarrhea (**Figure 6E**), stomatitis (**Figure 6F**), fatigue (**Figure 6G**), sensory neuropathy (**Figure 6H**), hand-foot syndrome (**Figure 6I**), and anorexia (**Figure 6J**). One study reported two fatalities in both the experimental and control groups due to neutropenia, although this finding was not statistically significant. The combined relative risk (RR) from all studies is shown in the figure. The results indicate that the incidence of grade 3–4 neutropenia, diarrhea, and neurologic disorders was significantly higher in the oxaliplatin treatment group compared to the control group, with statistical significance. Other outcome measures showed an increased risk, but no significant differences were observed.

## Discussion

In this meta-analysis, all seven included high-quality studies are prospective randomized controlled trials. Among them, two studies involve combination therapy with irinotecan, two with bevacizumab, and one with panitumumab. The use of oxaliplatin-based combination therapy as first-line treatment in elderly patients with metastatic colorectal cancer (mCRC) is associated with an improved ORR. Additionally, the analysis highlights the characteristics of grade 3–4 treatment-associated toxicities (TATE), demonstrating a manageable safety profile.

The benefits of adding oxaliplatin in adjuvant therapy for colorectal cancer are well-established; however, several factors may limit its utility in elderly patients. Age, especially when compounded by comorbidities, can make treatment more challenging in this population (30). In this meta-analysis, oxaliplatin-based chemotherapy regimens were found to significantly improve ORR, CR, and PR rates, allowing patients to benefit from increased tumor shrinkage or even remission. A higher ORR is often associated with faster symptom relief, which is particularly beneficial for patients experiencing severe symptoms. Tumor reduction can alleviate disease burden and improve symptoms such as pain and hematochezia, ultimately enhancing the quality of life for these patients. These findings are crucial for informing clinical decision-making and have significant practical implications for improving outcomes and quality of life in mCRC patients.

The results note that oxaliplatin did not significantly extend overall survival (OS) or progression-free survival (PFS). However, the results indicate a trend toward improvement in both OS (HR 0.97, 95% CI 0.86–1.08) and PFS (HR 0.90, 95% CI 0.79–1.01). Although these results are not statistically significant, they may still hold clinical relevance. The observed trends suggest that oxaliplatin-based therapy could provide a modest survival benefit, particularly in specific patient subgroups or when combined with targeted agents. For instance, the TRIBE2 trial demonstrated that intensified oxaliplatin-based regimens (e.g., FOLFOXIRI plus bevacizumab) significantly improved OS and PFS in patients with metastatic colorectal cancer (mCRC), particularly those with left-sided tumors, highlighting the importance of patient selection and combination strategies (19). Similarly, a meta-analysis by Cremolini et al. found that oxaliplatin-based chemotherapy was associated with improved survival outcomes in RAS wild-type mCRC patients, further supporting the potential clinical relevance of these trends (31). While the current analysis may lack statistical power to detect significant differences, larger randomized controlled trials (RCTs) with well-defined patient populations and standardized treatment protocols could potentially confirm these benefits. For example, the OPTIMOX trials explored intermittent oxaliplatin dosing to reduce toxicity while maintaining efficacy, demonstrating that tailored approaches could enhance the therapeutic index of oxaliplatin in elderly or frail patients (32). These findings underscore the importance of considering both statistical significance and clinical relevance when interpreting survival outcomes, as even modest improvements in OS or PFS can translate into meaningful benefits for patients, particularly in the context of personalized treatment strategies.

Although the results for OS and PFS did not reach statistical significance, oxaliplatin-based combination therapy demonstrated significant improvements in ORR, CR, PR, and DCR. These findings suggest that while oxaliplatin may not confer a survival advantage, it effectively enhances tumor response rates, which can translate into meaningful clinical benefits such as symptom relief and improved quality of life.(QoL). The improvement in ORR, CR, and PR indicates a higher likelihood of tumor shrinkage or remission, which is particularly valuable for patients with symptomatic disease or those seeking to downstage tumors for potential surgical resection. Furthermore, the increase in DCR suggests that oxaliplatin-based therapy can provide sustained disease stabilization, even in the absence of significant survival benefits. These response metrics are critical in evaluating the therapeutic efficacy of oxaliplatin, particularly in elderly or frail patients where maintaining QoL and controlling disease progression are prioritized over aggressive survival gains. This discrepancy between response rates and survival outcomes can be attributed to a variety of factors, including tumor biology, mechanisms of drug resistance, patient comorbidities, immune microenvironment dynamics, and pharmacogenomic variability. Tumor heterogeneity is a defining feature of metastatic colorectal cancer (mCRC), characterized by distinct subpopulations of tumor cells with diverse genetic, epigenetic, and phenotypic profiles (33). Oxaliplatin induces DNA damage and apoptosis in rapidly dividing cells, effectively targeting sensitive subclones and leading to significant tumor shrinkage, as reflected in improved ORR, CR, and PR. However, resistant subclones often survive and eventually dominate, driving disease progression and limiting long-term survival benefits (34). Recent studies suggest that cancer stem cells (CSCs) play a key role in this process, as they are inherently resistant to chemotherapy and capable of repopulating the tumor (35). The persistence of CSCs following oxaliplatin treatment may explain the disconnect between short-term response rates and long-term survival outcomes.

Oxaliplatin induces apoptosis by forming DNA adducts, but its efficacy is limited by various resistance mechanisms, including enhanced DNA repair, upregulation of drug efflux pumps, and alterations in apoptotic pathways (36). For instance, the upregulation of nucleotide excision repair (NER) pathways can repair oxaliplatin-induced DNA damage, gradually reducing the drug’s efficacy (37). Similarly, the overexpression of drug efflux pumps, such as ATP-binding cassette (ABC) transporters, lowers intracellular oxaliplatin concentrations, further contributing to resistance (38). Epigenetic modifications, including DNA methylation and histone acetylation, also play a role by altering the expression of genes involved in drug metabolism and cell survival (39). Collectively, these resistance mechanisms lead to the eventual failure of oxaliplatin-based therapy, despite initial improvements in ORR, CR, and PR.

The tumor immune microenvironment plays a crucial role in shaping the effectiveness of chemotherapy. Oxaliplatin has been shown to trigger immunogenic cell death (ICD), enhancing anti-tumor immune responses by releasing damage-associated molecular patterns (DAMPs) and promoting cytotoxic T cell infiltration (40). However, in mCRC, an immunosuppressive microenvironment—marked by the presence of regulatory T cells (Tregs), myeloid-derived suppressor cells (MDSCs), and immune checkpoint molecules—can counteract these effects (41). While oxaliplatin may initially boost response rates through ICD induction, this immunosuppressive milieu can limit the durability of its effects, hindering long-term survival benefits. Emerging evidence suggests that combining oxaliplatin with immune checkpoint inhibitors may help overcome these limitations, though further research is needed to confirm its clinical efficacy (42).

Patient-specific factors, including comorbidities and pharmacogenomic variability, can significantly impact the effectiveness and tolerability of oxaliplatin-based therapy. For example, patients with pre-existing liver dysfunction or renal impairment may be more susceptible to toxicity, often requiring dose adjustments or treatment discontinuation (7). Additionally, genetic polymorphisms in drug metabolism pathways, such as glutathione S-transferases (GSTs) and UDP-glucuronosyltransferases (UGTs), can influence oxaliplatin pharmacokinetics and toxicity, potentially leading to suboptimal drug exposure and reduced treatment efficacy (43). Moreover, comorbid conditions like diabetes, cardiovascular disease, and chronic inflammation can exacerbate treatment-related adverse effects, further compromising patient tolerance and long-term outcomes (44).

Tumor evolution under selective pressure from chemotherapy represents a key mechanism underlying the discrepancy between short-term response rates and long-term survival outcomes. Oxaliplatin-based therapy exerts selective pressure on tumor populations, promoting the emergence of resistant clones with distinct genetic and phenotypic characteristics. Liquid biopsy studies have shown that chemotherapy, including oxaliplatin, can drive the expansion of pre-existing minor resistant subclones that were initially present at low frequencies. For example, Siravegna et al. (45) explored clonal evolution in colorectal cancer patients undergoing treatment, demonstrating that resistant subpopulations emerge under therapeutic pressure, particularly in response to EGFR inhibitors and oxaliplatin-based chemotherapy. These findings underscore that dynamic shifts in tumor clonal architecture, driven by the selection of resistant clones, are a critical factor in the development of acquired resistance and subsequent disease progression. Moreover, recent studies have highlighted that the evolutionary dynamics of colorectal cancer under chemotherapy are significantly influenced by the tumor’s capacity to adapt through both genetic and epigenetic alterations. For instance, Tie et al. (46) employed circulating tumor DNA (ctDNA) analysis to track clonal evolution in patients with stage II/III colorectal cancer, demonstrating that chemotherapy selectively promotes the expansion of resistant subclones. These subclones acquire additional mutations that confer enhanced survival and proliferative advantages, allowing them to persist and proliferate despite ongoing treatment. This adaptive process ultimately drives tumor recurrence and progression, providing an explanation for why initial therapeutic responses to oxaliplatin—reflected in improved ORR, CR, and PR—fail to translate into durable survival benefits, such as prolonged OS or PFS.

Although oxaliplatin-based therapy may not improve OS or PFS, the observed enhancements in ORR, CR, PR carry significant clinical implications. High response rates can enable surgical resection of tumors previously deemed inoperable, offering the potential for a curative approach in select patients (47). Furthermore, achieving CR or PR can enhance quality of life by alleviating tumor-related symptoms, such as pain, obstruction, and bleeding (48). Additionally, the improvement in DCR suggests that oxaliplatin-based therapy may provide meaningful disease control, even if it does not result in a direct survival benefit.

The reduction in heterogeneity observed following subgroup analysis based on chemotherapy regimens underscores the critical impact of treatment combinations on disease control rate (DCR) in metastatic colorectal cancer (mCRC). In particular, the integration of targeted therapies, such as bevacizumab or panitumumab, with oxaliplatin-based chemotherapy may account for the variability in DCR outcomes. Studies have demonstrated that the addition of anti-angiogenic or anti-EGFR agents enhances the efficacy of cytotoxic chemotherapy by modulating distinct molecular pathways (19). However, the persistent heterogeneity in DCR highlights the influence of patient-specific factors, including tumor biology and molecular subtypes, on treatment response. For instance, patients with RAS wild-type tumors may derive greater benefit from EGFR inhibitors, whereas those with VEGF-driven angiogenesis are more likely to respond to bevacizumab (48). These findings emphasize the necessity of personalized treatment strategies in mCRC, guided by molecular profiling and biomarker-driven therapy, to optimize disease control and minimize variability in clinical outcomes.

Oxaliplatin is widely used to treat various malignancies, including mCRC (49). While oxaliplatin demonstrates significant clinical efficacy, TEAEs are relatively common, particularly in elderly patients, necessitating close monitoring and management. Adverse events reported in studies on mCRC patients were primarily grade 1 and grade 2, with fewer cases of grade 3 and grade 4 events. Common adverse events included hematologic toxicity, gastrointestinal symptoms, and neurological disorders (50). Other adverse reactions such as fatigue and neurogenic anorexia were also observed. Patients receiving oxaliplatin-based combination therapy had a higher incidence of grade 3–4 neutropenia, diarrhea, and neurological disorders compared to the control group. Although other endpoints indicated a potential increase in risk, the differences were not statistically significant and remain inconclusive. Additionally, there was no statistical difference in neutropenia-related mortality between treatment regimens.

For elderly patients, the safety profile of oxaliplatin requires particular attention. This population often has a higher incidence of comorbidities, decreased organ function, and reduced metabolic and excretory capacities, making them more susceptible to adverse drug reactions, which can impact both quality of life and treatment adherence (51). The incorporation of oxaliplatin-based combination therapy as a first-line treatment for mCRC involves a complex risk-benefit assessment, particularly in elderly patients, who represent a significant proportion of the affected population. These toxicities can profoundly impact QoL and limit the therapeutic benefits of oxaliplatin in this vulnerable population. The physiological characteristics of aging—including impaired renal and hepatic function, immunosenescence, and cumulative organ damage—profoundly influence the pharmacokinetics and toxicity profile of oxaliplatin. For instance, neurotoxicity, the dose-limiting toxicity of oxaliplatin, occurs in approximately 15–20% of elderly patients, often necessitating dose reductions or treatment discontinuation, which may compromise therapeutic efficacy (52). Additionally, polypharmacy-driven drug interactions further exacerbate treatment-related risks. Concomitant use of nephrotoxic agents, such as nonsteroidal anti-inflammatory drugs (NSAIDs), can potentiate oxaliplatin-induced renal dysfunction, while anticoagulants combined with bevacizumab may increase the risk of bleeding (53).These challenges underscore the critical role of comprehensive geriatric assessment (CGA) in risk stratification. The Cancer and Aging Research Group (CARG) toxicity score and the Chemotherapy Risk Assessment Scale for High-Age Patients (CRASH) model have demonstrated utility in predicting severe toxicity; however, prospective validation in elderly mCRC cohorts receiving oxaliplatin remains limited (14). Emerging evidence from the PRODIGE 34 trial (NCT04262687) suggests that CGA-guided dose adjustments can preserve QoL without compromising survival, advocating for a personalized rather than regimen-driven approach to treatment (37).

In our analysis, the observed dissociation between improvements in ORR and DCR versus the stagnation of OS likely reflects the dual impact of tumor heterogeneity and acquired resistance. While oxaliplatin effectively targets proliferative tumor subclones, residual resistant populations—enriched with stem-like or slow-cycling cells—can drive disease progression (19). Single-cell sequencing studies have demonstrated that chemotherapy exerts selective pressure, favoring clones with enhanced DNA repair capacity (e.g., ERCC1 overexpression) or apoptotic resistance mechanisms (e.g., BCL-2 upregulation), underscoring the need for sequential or combination strategies to address clonal evolution (54).However, in elderly patients, the cumulative toxicity of intensive combination regimens often limits their feasibility. The GO2 trial demonstrated that lower-intensity chemotherapy (e.g., 60% of the standard dose) yielded comparable survival outcomes to full-dose treatment while significantly improving quality of life in frail patients, a paradigm that may be applicable to oxaliplatin-based regimens (46). Mohile et al. demonstrated in a study of 718 patients that conducting a geriatric assessment (GA) and implementing appropriate interventions before initiating chemotherapy significantly reduced the risk of severe toxicity from cancer treatment (55). This approach allows clinicians to identify patients at higher risk of adverse events and tailor treatment regimens accordingly, potentially improving both safety and efficacy. Furthermore, dose modifications, supportive care measures, and close monitoring should be prioritized to mitigate toxicity and enhance treatment adherence.

To address the existing evidence gaps and optimize oxaliplatin-based therapy for elderly patients with metastatic colorectal cancer (mCRC), several key areas warrant further investigation. First, large-scale prospective studies are needed to validate geriatric assessment (GA)-based algorithms for oxaliplatin dosing. The ongoing GERICO trial (NCT04961450) exemplifies this approach by integrating frailty biomarkers (e.g., interleukin-6, gait speed) with clinical parameters to predict toxicity and enable dynamic dose adjustments (56). Such efforts could refine risk stratification and improve treatment tolerability in this vulnerable population. Second, biomarker-driven stratification holds promise for identifying subsets of elderly patients most likely to benefit from oxaliplatin-based therapy. Tumor molecular profiling and liquid biopsy-based monitoring (e.g., circulating tumor DNA [ctDNA] dynamics) could guide personalized treatment decisions by predicting response to specific therapies. For instance, low expression of ERCC1, a key enzyme in the nucleotide excision repair pathway, has been associated with increased sensitivity to oxaliplatin, suggesting its potential as a predictive biomarker for chemotherapy efficacy (57). Beyond chemotherapy, biomarker-driven approaches are crucial for optimizing targeted therapies. The BEACON CRC trial demonstrated that BRAF V600E mutations confer resistance to EGFR inhibitors, such as cetuximab, while highlighting the efficacy of combinatorial BRAF/MEK/EGFR inhibition in overcoming this resistance (58). These findings underscore the critical role of molecular profiling in clinical decision-making, facilitating the development of precision medicine strategies tailored to individual tumor biology and patient-specific characteristics. Third, future clinical trials should prioritize patient-reported outcomes (PROs) as primary endpoints to better align therapeutic goals with patient priorities. The NCI PRO-CTCAE tool has been widely adopted to measure symptomatic adverse events, providing valuable insights into the patient experience and guiding supportive care interventions (59). This shift in focus could lead to more patient-centered care and improved treatment adherence. Finally, innovative therapeutic strategies are needed to balance efficacy and toxicity. Intermittent therapy, as explored in the OPTIMIZE trial (NCT03768222), aims to mitigate cumulative neurotoxicity through “chemo-holidays” while maintaining disease control (60). These approaches could reduce treatment-related toxicity and improve outcomes in elderly mCRC patients. Current research primarily focuses on the combined use of oxaliplatin with other agents. However, for metastatic colorectal cancer, particularly in patients with proficient mismatch repair (pMMR)/microsatellite-stable (MSS) colorectal cancer, standard treatments have shown suboptimal efficacy, and the benefit of combining these treatments with immunotherapy remains uncertain. Some studies have indicated that short-course radiotherapy combined with CAPOX and the PD-1 inhibitor serplulimab can achieve improved pathological complete response (pCR) rates in locally advanced pMMR/MSS colorectal cancer (61). In a study by Wang et al., although median OS was not reached with serplulimab in combination with HLX04 and XELOX, a trend toward improved OS was observed, suggesting promising therapeutic potential (62). Additionally, research has explored the use of HSPD1 inhibitors to enhance the cytotoxicity of oxaliplatin (63), potentially increasing its therapeutic efficacy. With advancements in circulating tumor DNA (ctDNA) research, precision adjuvant therapy may lead to improved outcomes for elderly patients with metastatic colorectal cancer.

This study has several limitations that warrant consideration. First, substantial heterogeneity was observed in the disease control rate (DCR), which may be partly attributed to variations in age distribution across the included studies. While two studies had a median patient age above 65 years, they also included a subset of younger patients, potentially confounding the overall findings. Although age was not identified as an independent prognostic factor, its potential influence on treatment outcomes cannot be entirely excluded. Second, the presence of comorbidities in elderly patients, a key determinant of treatment tolerability and efficacy, was not comprehensively reported in the included studies. The absence of detailed comorbidity data limits our ability to assess their impact on clinical outcomes, particularly in terms of treatment-related toxicity. Third, variability in chemotherapy regimens—including oxaliplatin-based combinations with bevacizumab, panitumumab, or irinotecan—may have contributed to the observed heterogeneity, as different therapeutic combinations can elicit distinct responses. Finally, the lack of specific molecular data, such as microsatellite instability (MSI) status and RAS mutations, precluded biomarker-driven subgroup analyses. Given the growing recognition of molecular heterogeneity in metastatic colorectal cancer, future studies should incorporate a broader spectrum of clinical and genomic parameters to refine patient stratification and optimize therapeutic decision-making in elderly patients receiving oxaliplatin-based combination therapies.

## Conclusion

In conclusion, oxaliplatin-based first-line treatment in elderly patients with mCRC demonstrates significant advantages in terms of ORR, CR, PR. However, it does not show superiority in OS or PFS. While toxicity is generally manageable, the high incidence of adverse effects warrants careful consideration, particularly in elderly patients. Oxaliplatin-based regimens may be considered for patients with high tumor burden who urgently require tumor reduction and symptom relief, following a comprehensive evaluation.

## Data Availability

The data used to support the findings of this study are available from the corresponding author upon request.
